# Traffic monitoring system design considering multi-hazard disaster risks

**DOI:** 10.1038/s41598-023-32086-6

**Published:** 2023-03-25

**Authors:** Michele Gazzea, Amir Miraki, Onur Alisan, Monique M. Kuglitsch, Ivanka Pelivan, Eren Erman Ozguven, Reza Arghandeh

**Affiliations:** 1 Department of Computer Science, Electrical Engineering and Mathematical Sciences, Western Norway University of Applied Sciences, 5063 Bergen, Norway; 2 Department of Civil and Environmental Engineering, Florida A & M University-Florida State University College of Engineering, Tallahassee, 32310 USA; 3 Fraunhofer Institute for Telecommunications, Heinrich Hertz Institute, HHI, 10587 Berlin, Germany

**Keywords:** Environmental sciences, Natural hazards, Engineering

## Abstract

Roadways are critical infrastructure in our society, providing services for people through and between cities. However, they are prone to closures and disruptions, especially after extreme weather events like hurricanes. At the same time, traffic flow data are a fundamental type of information for any transportation system. In this paper, we tackle the problem of traffic sensor placement on roadways to address two tasks at the same time. The first task is traffic data estimation in ordinary situations, which is vital for traffic monitoring and city planning. We design a graph-based method to estimate traffic flow on roads where sensors are not present. The second one is enhanced observability of roadways in case of extreme weather events. We propose a satellite-based multi-domain risk assessment to locate roads at high risk of closures. Vegetation and flood hazards are taken into account. We formalize the problem as a search method over the network to suggest the minimum number and location of traffic sensors to place while maximizing the traffic estimation capabilities and observability of the risky areas of a city.

## Introduction

Extreme weather and climate events have increased in frequency or magnitude in recent decades. Likewise, populations and assets at risk have also increased, with higher consequences for exposed and vulnerable infrastructure systems. Roadways are one of the most critical types of infrastructure in our society. They allow the movement of people, goods, and services through and between cities, improving the quality of life in a populated area. Our daily responsibilities heavily depend on the performance of the transportation system. Therefore, efficiently operating and maintaining it becomes crucial for mobility and the sustainability of human life.

Although there is tended to think natural disasters’ impacts on roadways are distinct circumstances, all too often, they are a complex series of events that stretch over and build on other catastrophes such as storms, floods, fires, structural collapse, etc^1^. Nevertheless, opportunities for managing compound risks of weather- and climate-related disasters can be developed by roadway authorities for effectively managing multi-hazard risks and adapting to climate change, including adjustments to current roadway monitoring systems.

Traffic flow data are one of the most fundamental types of information for any transportation system. Roadway authorities place sensors at specific locations along the roads to measure the distribution and variation of traffic and calculate the annual average daily traffic (AADT). AADT is used for analyzing accident rates, highway planning, designing arterial street systems, estimating mobility trends, determining roadway geometry, congestion management, pavement design, etc.^2^.

Due to the exponential growth in the built environment and the length of roads, it has become increasingly difficult and costly to place sensors on all roads. In addition to the high installation cost, traffic sensors have high operating and maintenance costs^3^. To achieve cost efficiency, researchers have proposed some optimal and near-optimal traffic sensor placement methods,^4–7^.

However, traffic monitoring systems are mostly designed for blue-sky days without considering emergency conditions after extreme weather and climate events. Therefore, cities are more vulnerable and disable to provide efficient evacuation strategies and restoration operations due to the lack of observability of critical sections of the roadway systems.

Natural disaster risk assessment for roadways is widely addressed in the literature, e.g.^8–10^. Traditionally, risk maps of the cities are created via visual inspection by the ground base patrols and sometimes using drones or helicopters. These procedures are, however, time consuming and costly. Recently, satellites have become a viable solution for large-scale applications, especially for urban infrastructure assets^11^. The advantage of satellites comes from the optimal trade-off between resolution (up to 0.3 meters/pixel), quality, revisiting time, and cost. This allows efficient and frequent monitoring and better situational awareness about the status of roadways at the city level. These paper’s authors have developed machine learning-based approaches to detect roadway closures automatically after storms using high-resolution satellite images^12–14^.

In this paper, we tackle the problem of optimal sensor placement on roadways to address two tasks simultaneously. The first task is traffic data estimation in normal situations. The second one is to have enhanced observability of roadways in case of extreme weather events in high-risk areas. We propose a satellite-based multi-domain risk assessment to locate roads at high risk of closures, given the vegetation along roadways and the possibility of floods. Furthermore, we design a novel graph-based method to estimate traffic flow on roads where sensors are not present. Finally, we formalize the problem as an iterative search method over the graph to minimize the number of sensors while maximizing the estimation capabilities and observability of the city.

New roadway monitoring systems should be planned and designed to account for compound risks due to the climate changes that may occur over their lifetimes. In addition, existing roadway monitoring systems may need to be retrofitted, given climate change. Yet, decision-makers need access to high-quality and reliable traffic information to support technical and institutional capacity to manage climate-related risks. To summarize, this paper’s contributions are as follows:

1. to create a multi-hazard risk assessment framework using satellite-based AI and
2. to develop a multi-objective optimization scheme for traffic sensor placement considering normal and emergency conditions

## Use case

The study area is the city of Tallahassee, the capital of Florida. Several tropical cyclones severely hit Tallahassee in the last years including hurricanes of Category 3 or above. These include Tropical Storm Debby (2012), Hurricane Hermine (2016), Hurricane Michael (2018), and Hurricane Ian (2022). During Hurricane Michael, 90% of the city-county remained without power for up to a week, and numerous fallen trees throughout the community made commuting almost impossible^15^.

For our approach, we acquired the data partly from the municipality, partly from the public domain, and commercial satellite images to develop our approach. In particular, we acquired a satellite image of the entire city from the PlanetScope constellation^16^, covering 402.8 $\text {km}^2$ (155.5 $\text {mi}^2$). The image contains eight spectral bands (coastal blue, blue, green I, green II, yellow, red, red edge, and near-infrared) with a spatial resolution of 3 m/pixel.

**Figure 1:**
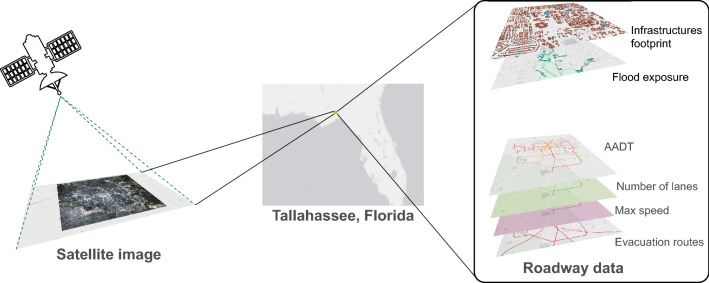
Overview of the study area and the data used. The satellite image has been acquired from the PlanetScope constellation^16^. The satellite image and the roadway data are visualized and integrated with QGIS 3.20^17^.

Additionally, we used Geographical Information System (GIS) data, including shapefiles of the roads storing their geographical coordinates, number of lanes, maximum speed limit, and the annual average daily traffic (AADT) traffic data. The locations of all the buildings (houses, private or public facilities, etc.) are also acquired. Moreover, a subset consisting of hospitals, medical facilities, fire stations and emergency shelters is extracted. We refer to these buildings as *critical buildings* as this kind of building becomes extremely important during disaster management. Finally, locations of high probability of flood were acquired from the municipality. Such areas have been calculated according to the past flooding history and include primarily lakes and rivers as well as and wetlands such as swamps and marshes. Figure 1 summarizes the data used.

## Methodology

In this paper, we are interested in optimizing the number of traffic sensors for urban roadway systems. The sensor network should have high traffic prediction capabilities. This means that it is possible to estimate the traffic flow on roads that do not have sensors based on the data of the surrounding streets. At the same time, such sensors can serve as an additional surveillance tool, especially for disaster management, to have better situational awareness.

**Figure 2:**
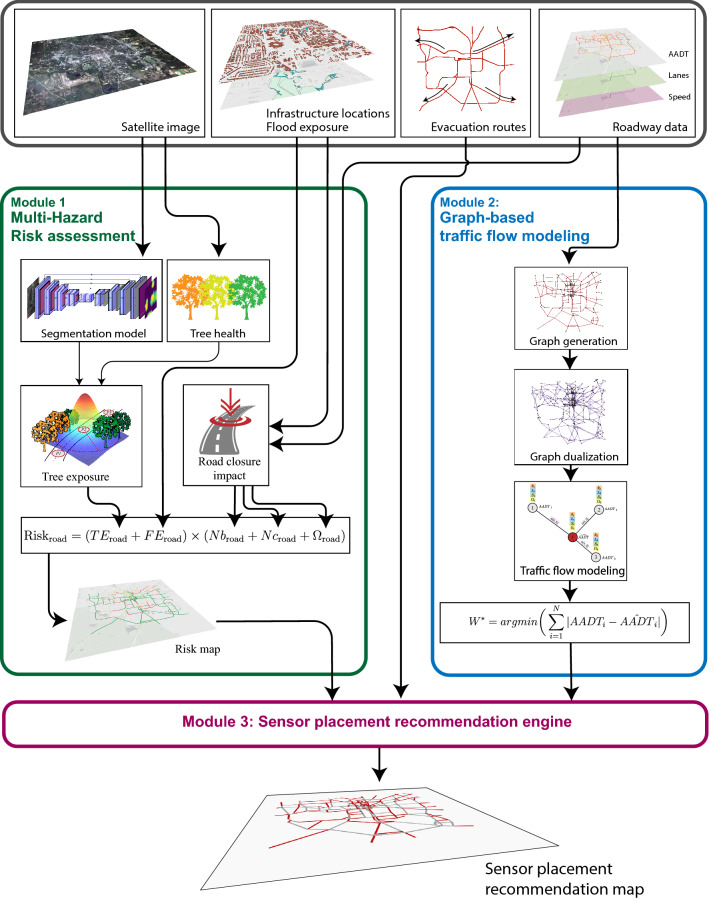
Pipeline of the proposed approach. Module 1 computes risky areas prior to a natural disaster. Module 2 models and predict the traffic flow over the roads. The two modules are run in parallel. Finally, the outcomes from the two modules are used in Module 3 to provide an optimal sensor placement that can maximize the traffic prediction performance while covering the at-risk areas with the minimum number of sensors. QGIS 3.20^17^ software was used to create the maps, integrate and plot all the data and results.

Given these premises, our framework is composed of two main modules. In the first module, we use a satellite image and available GIS data to create a risk map assessment of the roadway infrastructure automatically. Risk locations are essential for monitoring prior to extreme events. In the second module, we use a tailored graph-based modeling algorithm to estimate road traffic flow. This is related to traffic forecasts in ordinary situations. Finally, we combine the two outcomes in a multi-objective recommendation engine to suggest a sensor placement map. In other words, we want to place the sensors in locations that improve observability and situational awareness of the at-risk areas while providing good estimation capabilities of the traffic on other roads. Figure 2 sketches our proposed framework, and each component is further described in the following.

### Module 1: Multi-hazard risk assessment

 The risk of an event is, by definition^18^, the product between the likelihood *L* of that event to occur and the impact *I* on the society or environment (Eq. 1).

1 $$\begin{aligned} \text {Risk} = L \otimes I \end{aligned}$$

Equation (1) states that a harmful event, even if it has a high probability to occur, can still be categorized as low risk if it does not have significant consequences on the society or environment.

In this study, we are considering the road network disruption after a hurricane. From the literature^19^ and our experience, we know that trees (e.g., fallen trees, tree debris, etc.) and floods are some of the leading causes of roadway closure and damages following extreme weather events, like hurricanes. A mathematical formulation of the likelihood $\mathcal {L}$ as a probabilistic function is challenging to derive due to the stochastic nature of hurricanes. Some studies, such as^20^ and^21^, tried to quantify single tree failure probability due to extreme weather events by proposing empirical mechanical models to estimate the possibility of tree failures. Such works consider tree characteristics (e.g., tree canopy, stem mass and tree mass, diameter at breast height, etc.), soil strength, and wind-induced bending moment on each tree and combine them with wind data^22^. However, due to the complexity and limitations of measuring such characteristics, which are required information for mechanistic models, it is often impossible to obtain accurate estimations of the failure probability for trees on a large scale. Statistical models are another approach for predicting the probability of wind-related tree failure, as shown in^23^, but they still require data from surveys and inventories. Accurate flood predictions also require several types of data, such as the amount of rainfall occurring on a real-time basis, the rate of change in river stage, knowledge about the type of storm producing the moisture (duration, intensity and areal extent) and about the characteristics of rivers’ drainage basin, such as soil-moisture conditions, topography, and impermeable land area^24^. Therefore, in this study, we propose a simplified calculation based on tailored vegetation and flood exposures.

The impact of a roadway closure is, on the other hand, directly connected to the consequences of such a closure on the transportation network. It depends on the number of citizens and activities affected by the closure, whether it serves critical buildings (e.g., hospitals and emergency stations) and the importance of the road itself for the connectivity of the network. As such, we can rewrite Eq. (1) for our use case as:

2 $$\begin{aligned} \text {Risk} = \underbrace{\text {Tree Exposure; Flood Exposure}}_{L} \otimes \underbrace{\text {(Building Density; Building Importance; Road Importance)}}_{I} \end{aligned}$$

Therefore, in this first module, we detect at-risk areas within the transportation network by assessing the trees using satellite images. We assign a tree exposure score to each road based on the quantity, density, health, and distance of trees to the road. Furthermore, we use the areas that are more prone to be flooded to compute flood exposure per each road. ’ Then, we integrate the urban data from the municipality, precisely the number of buildings along the road, emergency facilities, and roadway locations. Finally, tree exposure, flood exposure, and impact are combined into a risk evaluation using Eq. (2). The output is a closure risk map of the road network.

#### Trees segmentation

We use remote sensing techniques based on satellite images to detect trees, which are the main cause of roadway closures after hurricanes. Trees pose a significant threat as they can easily fall on the roads due primarily to the impact of strong winds. Furthermore, locations with an increased number of trees are also dangerous as it is more likely that some of them may fall, as experienced during Hurricanes Hermine and Michael^25^. It would be extremely challenging to acquire single tree parameters (e.g., height, canopy size, stem diameter) from satellite images, especially given the 3m/px image resolution. Nevertheless, we can calculate the density of trees, their distances from the road and estimate their health, which are the essential factors to consider for the proposed closure risk analysis.

**Figure 3:**
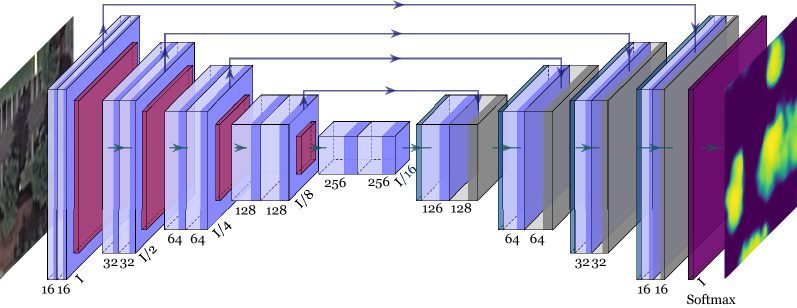
Deep learning model architecture used to segment vegetation from a satellite image. Given a satellite image *I* as input, the corresponding output is a mask where trees are detected. Convolutional block (blue), pooling layers (red), up-sampling layers (blue), concatenation layers (gray), and classifier (purple).

We first design a tree segmentation model $M^{tree}$. Given an input image, the corresponding output is a binary pixel-wise mask $O^{tree}$ taking two values, 0 for *no-trees-bearing* pixels and 1 for *trees*-bearing pixels. The segmentation model labels the pixels that belong to or are part of a tree. We use an encoder-decoder-based U-net architecture^26^ as a segmentation model, tailoring it for our application. The architecture is composed by a cascade of [16, 32, 64, 128, 256] convolutional filters activated by a *relu* activation function, followed by a batch normalization layer and a Max Pooling layer. The architecture is shown in Fig. 3. Binary cross entropy is used as loss function for training the network since only two labels are considered.

#### Trees’ health estimation

Another element to consider is the tree health. An unhealthy tree may be an indication of a pathogenic wood-induced decay. As such the tree has a higher general propensity to fail^27^. The health of a tree is generally connected to the quantity and quality of its foliage, which is related to the amount of chlorophyll pigment in the leaves. However, a low amount of chlorophyll is not necessarily connected to poor health. Low chlorophyll content can be due to, for instance, species that are naturally less green than others or natural seasonal phenological cycles. Tree type is another factor that can affect the tree failure as some species are more susceptible than others to uprooting^28^. However, estimating tree species from satellites is a challenging task. Some studies, like^29^, suggest an approach enabling a tree segmentation model to classify tree species. However, it requires at least a tree species reference dataset, which is not available in our study and is seldom available in general. Nevertheless, some studies, for example^30^, suggest that monitoring long-term trend in vegetation indices can give valuable information about a tree’s status. Positive trends are associated with growth while negative trends with decay in the tree foliage, thus health situation.

We use the Green Chlorophyll Vegetation Index (GCVI) calculated from the multi-spectral satellite as:

3 $$\begin{aligned} GCVI = \bigg ( \frac{\rho _{NIR}}{\rho _{GREEN}} \bigg )-1 \end{aligned}$$

where $\rho _{NIR}$ and $\rho _{GREEN}$ are the atmospheric reflectance for the infrared and green band, respectively. The GCVI is used to estimate leaf chlorophyll content across a wide range of plant species. It provides a good prediction of chlorophyll content while allowing for more sensitivity and a higher signal-to-noise ratio than other indexes^31^. Common applications include monitoring the impact of seasonality, environmental stresses, and vegetation health.

To compute the trend of the vegetation index, we calculate the GCVI difference $\Delta _{GCVI}$ for the same area in two consecutive years $t=2022$ and $t-1=2021$ as: $\Delta _{GCVI} = GCVI_t - GCVI_{t-1}$. Both the images were acquired between June and July, which is summer in Tallahassee, with little variation in phenology among the tree species. However, it is worth mentioning that, in general, the time interval heavily depends on the study area. In high temperate areas with more phenological variations among the species, additional time frames should be acquired, as explained in^30^. We expect the distribution $\Delta _{GCVI}$ for the trees in the city to follow a Gaussian-like distribution. Practically, this means that most of the trees have been neutral over the two years. A minority of them experienced a positive $\Delta _{GCVI}$ trend (growth) and others a negative $\Delta _{GCVI}$ trend (decay). To isolate the most significant negative trend in $\Delta _{GCVI}$, similarly to^19^, we empirically set a threshold *Th* as $Th = \mu - \sigma$, where $\mu$ and $\sigma$ are the mean and standard deviation of the $\Delta _{GCVI}$ distribution. This is justified by the fact that we are interested in detecting changes in the vegetation index that are statistically significant in relation to the average changes in the whole area. If a Gaussian model is representative of the empirical data, this threshold is considered reasonable by the authors.

Finally, we introduce a function $f_{health} (\cdot )$ that maps the value of $\Delta _{GCVI}$ into a *health level factor* value $O_{health}$. Trees with a $\Delta _{GCVI} > Th$ are assigned a health level factor $''1''$ (healthy) and trees with $\Delta _{GCVI} < Th$ are assigned a health level factor 2 (unhealthy). Mathematically,

4 $$\begin{aligned} O_{health} = f_{health}(\Delta _{GCVI}) \end{aligned}$$

Vegetation indexes can estimate the amount of chlorophyll in vegetation. However, in general, they have issues discriminating between trees and other smaller types of vegetation (e.g., bushes, grass, or fields). Therefore, the final step is to multiply $O_{health}$ with the tree segmentation map $O^{tree}$ obtained from the segmentation model $M^{tree}$. This allows for filtering out grass and fields, keeping only trees into consideration. The final output, $O^{tree}_{health}$ defined as

5 $$\begin{aligned} O^{tree}_{health} = O_{health} \otimes O^{tree} \end{aligned}$$

is a pixel-wise mask covering the whole study area where each pixel has one of the following values: 0 (no-tree), 1 (healthy tree) or 2 (unhealthy tree).

#### Trees exposure calculation

**Figure 4:**
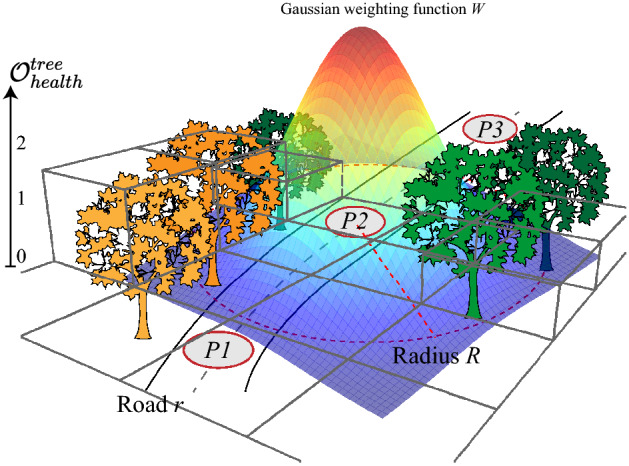
Tree exposure for a road. The road is sampled into equally distributed points $P_1, P_2, \dots P_n$. For each point we calculate tree exposure based on the amount and status of trees derived from $\mathcal {O}^{tree}_{health}$, weighted by a Gaussian-like function *W*.

Finally, we combine tree density, proximity, and health into a number called *tree exposure on road* *TE*. To compute *TE*, we first extract equally distributed points every 20 meters along the road. For each point *P*, we compute the tree exposure $TE_{P}$ by considering the health, distance from the roadway, and the density of the trees within a certain radius *R* from the point. We use a Gaussian weighting function to assign more weight to the trees closer to the road. The vegetation exposure calculation process is described in Eq. (6).

6 $$\begin{aligned} TE_{P} = \iint _{\varnothing _R} O^{tree}_{health} \cdot W \end{aligned}$$

where $O^{tree}_{health}$ is the map calculated in the previous subsection and *W* is a Gaussian weighing function, introduced to assign more importance to the pixels at the center of the road. The integral is calculated within a circle of *R* radius from the point *P* on the roadway. Figure 4 graphically illustrates the process.

Finally, the tree exposure for the entire road is defined as the sum value among all the vegetation exposure calculated at the different sampled points (Eq. 7).

7 $$\begin{aligned} TE_{\text {road}} = \sum _{\forall \text {P} \in \text {road}} TE_\text {P} \end{aligned}$$

We scale the value of the tree exposure for all the roads in the network within the range (0, 1).

#### Flood exposure

Similarly, we compute a number called flood exposure *FE* for each road. The flood exposure is calculated using the high flood probability zones provided by the municipality. Such zones are composed of water basins (e.g., rivers and lakes) and wetlands (e.g., marsh and swamps). We compute per each road the intersection between the road path and such high flood probability zones over the total road length. Hence:

8 $$\begin{aligned} FE_{road} = \text {road path} \cup \text {flood zone} \end{aligned}$$

A high value of flood exposure means that a road is passing through an area more prone to be flooded.

#### Roadway closure impact assessment

Each road provides an essential way of accessing the different buildings in a city. If a road is blocked because of a fallen tree or a flood, the closure isolates the buildings and infrastructures accessed from that road. Moreover, a road connects topologically and geographically two points in a city. If a road is blocked, it may disconnect parts of the network, especially if there are few other roads to bypass the closed section. We use the betweenness centrality for roads (i.e., edges in the graph)^32^ to measure the degree of redundancy of roads mathematically. For each road, we calculate the number of buildings $N_b$, the number of critical buildings $N_c$ that have access to that road and the measure of centrality $\Omega$:

9 $$\begin{aligned} Nb_\text {road} = \sum _{\text {road}} \text {buildings}; \qquad Nc_\text {road} = \sum _{\text {road}} \text {critical buildings}; \qquad \Omega _\text {road} = \sum _{s,t \in V} \frac{\sigma (s,t |road)}{\sigma (s,t)} \end{aligned}$$

where *V* is the set of nodes of the graph, $\sigma (s,t)$ is the number of shortest path between *s* and *t*, and $\sigma (s,t|r)$ is the number of those paths passing through road *r*. The number of buildings $N_b$, critical buildings $N_c$ and $\Omega$ are, similarly to the previous case, normalized within the range (0, 1).

#### Overall multi-hazard risk assessment

Finally, we combine the results form the previous calculations into a multi-hazard risk calculation. We compute the risk of a closure per each road by combining the tree exposure *TE* calculated from Eq. (7) and flood exposure *FE*, with the impact of disruption on the roadway network (Eq. 10). We recall that each term has been scaled within the same range (i.e., (0, 1)). This allows a direct sum without magnitude difference issues.

10 $$\begin{aligned} \text {Risk}_\text {road} = (TE_\text {road} + FE_\text {road}) \times (Nb_\text {road} + Nc_\text {road} + \Omega _\text {road}) \end{aligned}$$

### Module 2: Graph-based traffic flow modeling

From a mathematical view, a roadway network can be considered a graph where edges are roads and nodes are intersections between roads. Therefore, a transportation network can be analyzed using graph theory tools^33,34^. Graphs are one of the most important mathematical tools to model and analyze networks. In this paper, we consider the connected, undirected graph $G = (V, E)$, where *V* is the set of vertices (or nodes), and *E* is the set of edges. We say that two vertices *u* and *v* in a graph *G* are connected if *G* contains a path from *u* to *v*^35^. The set of all adjacent vertices of *v* in *G* is called the neighborhood of *v* and is denoted by *N*(*v*).

When working with a geographical network (i.e., nodes and edges must have geographical coordinates to locate them in a real-world reference system), a pre-processing simplification step is needed. Roads are usually composed of piece-wise linear segments and include additional nodes that only exist to help streets bend around curves. However, they are not nodes in a topological sense. Topological nodes consist only of intersections between roads. Therefore, we first run a simplification process that removes nodes having $N_G(v) = 2$.

After the simplification procedure, we consider the problem of estimating the traffic flow (i.e., AADT) of edges given the information of other edges. We recall that in the transportation graph, edges are roads. To ease the estimation procedure, we first calculate the dual graph out of the original graph. A dual graph $G'$ of a graph *G* is defined such that the edges of *G* are the nodes of $G'$ and the nodes of *G* become the edges of $G'$. Note that this operation is reversible. In our case, the dual graph of the considered transportation network represents roads as nodes and intersections as edges. This way, the traffic information, such as AADT, is encoded into nodes. In estimation problems, the missing information of a node $x_i$ is assumed to be a function *f* of the information of its neighborhood, such that $x_i = f(x_j), \; x_j\in N(i)$ Typically, the most commonly used function is an average of the information around its neighborhood:

11 $$\begin{aligned} x_i = \frac{1}{|N(i)|} \sum _{\forall j \in N(i)} x_j \end{aligned}$$

However, such a calculation may discard important information. Therefore, we propose a new estimation method for traffic data that considers a broader range of road characteristics and influences the traffic flow. We extract attributes for each road, specifically: the maximum speed limit *S*, number of lanes *L*, and network topology (orientation of the road $\theta$ and betweenness centrality $\Omega$.) The speed limit and number of lanes as taken from the roadways shapefile data. The centrality $\Omega$ is calculated based on the road graph according to Eq. (9). The orientation $\theta$ is instead calculated as $\arctan {\frac{\Delta y}{\Delta x}}$ for each road, where $\Delta x$ and $\Delta y$ is the difference between the ending point and the starting point of the road in the *x* and *y* axis, respectively.

To estimate the AADT on a node, we parameterized the function *f* as a linear combination of the AADT of its neighborhood. Mathematically,

12 $$\begin{aligned} \hat{AADT}_i = f(AADT_j, j \in N(i)) = \frac{\sum _{j \in N(i)} \alpha (i,j) AADT_j}{\sum _{j \in N(i)} \alpha (i,j)} \end{aligned}$$

where $\hat{AADT}_i$ is the estimated AADT value for the node *i*, $j \in N(i)$ denote all the nodes in the neighborhood of *i* and $\alpha (i,j)$ are numerical parameters. In addition, we write the parameters $\alpha (i,j)$ as a linear combination of the road features $\phi$:

13 $$\begin{aligned} \alpha (i,j) = W_1 \phi _\theta + W_2 \phi _L + W_3 \phi _S + W_4 \phi _\Omega \end{aligned}$$

where $W_1, W_2, W_3$ and $W_4$ are non-negative constants, acting as weights for the features. The road features $\phi _\theta , \phi _L, \phi _S$ and $\phi _\Omega$ are calculated from the road attributes $\theta , L,$ *S* and $\Omega$ as follows:

14 $$\begin{aligned} \alpha (i,j) = W_1 \underbrace{|\cos (\theta _i - \theta _j)|}_{\phi _\theta } + W_2 \underbrace{\bigg [ 1-\frac{|L_i - L_j|}{\max (L) - \min (L)} \bigg ]}_{\phi _L} + W_3 \underbrace{\bigg [ 1-\frac{|S_i - S_j|}{\max (S) - \min (S)} \bigg ]}_{\phi _S} + W_4 \underbrace{\bigg [ 1-\frac{|\Omega _i - \Omega _j|}{\max (\Omega ) - \min (\Omega )} \bigg ]}_{\phi _\Omega } \end{aligned}$$

**Figure 5:**
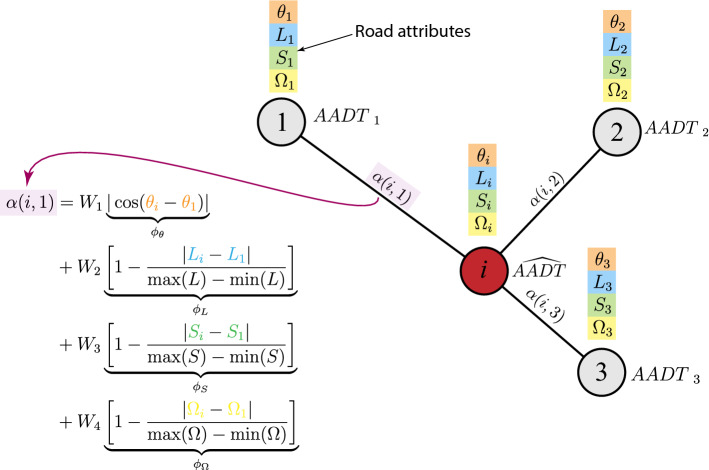
Node data estimation approach. The AADT value of the node *i* (red node) is estimated given the information provided by its neighborhood (here, nodes 1,2, and 3) weighted by the parameters $\alpha (i,j), j = 1,2,3$. The parameters $\alpha$ are calculated based on each node attribute: speed *S* (green), number of lanes *L* (blue), orientation $\theta$ (orange) and centrality $\Omega$ (yellow).

Figure 5 summarizes our node data estimation approach.

Finally, we solve the task of finding $W_1, W_2$ and $W_3$ as an optimization problem to minimize for the predicted traffic data (AADT) over the whole graph.

15 $$\begin{aligned} (W_1^*, W_2^*, W_3^*, W_4^*) = {{\,\mathrm{arg\,min}\,}}\bigg ( \sum _{i=1}^{N} |AADT_i - \hat{AADT}_i|\bigg ) \end{aligned}$$

where $(W_1^*, W_2^*, W_3^*, W_4^*)$ are the optimized weights.

### Module 3: Multi-objective recommendation

In the previous section, the optimal values of the parameters $W_1, W_2, W_3$ and $W_4$ are found such that the AADT estimation is minimized for the whole graph. Nevertheless, in our study, we are interested in uncovering the at-risk areas, minimizing the traffic estimation error and keeping the number of sensors deployed as low as possible. Because the risk value assigned to each road, using Eq. (10), is a non-negative continuous number, it is not straightforward to categorize a road as *at risk* or *not at risk*. Therefore, we use the maximum deviation method^36^ to calculate a threshold $Th_{risk}$ for the risk values. Roads with risk values less than such a threshold are categorized as *not at risk*, and roads with risk value greater or equal to the threshold are categorized as *at risk*. The maximum deviation method has been developed to find a threshold for uni-modal distributions where one group (in this case, not at risk roads) is larger than a second group (in this case, at risk roads). This way, roads are assigned to two groups: at-risk and not-at-risk roads. The multi-objective optimization scheme for traffic sensor placement is described in Algorithm 1.
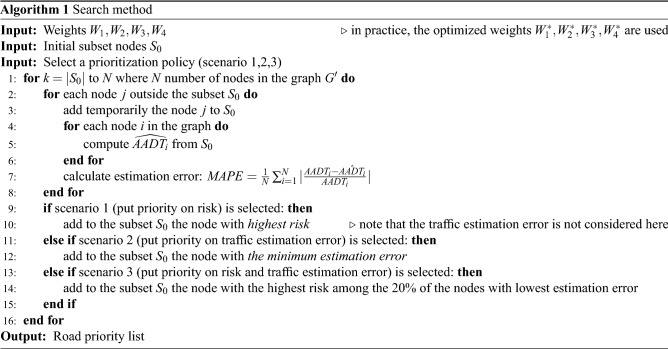


As a starting point for our sensor placement strategy, we assume that we need to monitor all the at-risk roads and evacuation routes around the city. We denote the union of at-risk roads and evacuation routes as $S_0$, which is the minimum set of roads to be monitored in any case. This indirectly sets a lower bound on the number of sensors to deploy. Then, we propose an iterative search method to find additional roads to monitor in case more sensors are available. The inputs of the search method are: (i) the initial set $S_0$ to monitor, (ii) the weights $W_1,W_2,W_3$ and $W_4$ modeling the traffic flow over the graph, as described in Eq. (13), and (iii) a prioritization policy. Although in practice, the optimal weights $W_1^*,W_2^*,W_3^*$ and $W_4^*$ found during the optimization step (Eq. 15) should be used, any other values of the coefficients are possible in theory. The output is a road priority list, which indicates the roads that should be prioritized and monitored by a sensor to ensure minimum traffic estimation error. We consider three different prioritization policies depending on whether, in adding more sensors to new roads, we prioritize: monitoring at-risk roads (scenario 1), having better traffic estimation capabilities (scenario 2), or both (scenario 3).

## Results and discussion

The framework has been developed in Python, using the Tensorflow/Keras libraries for the deep learning modules, scikit-image for the image processing parts, and networkx library for the topology analysis. QGIS 3.20 has been used to visualize and integrate all the different data. The testing platform is a computer equipped with an Intel i7 Core 10th Gen processor, 32GB of RAM, and an Nvidia RTX 2080 Super as a GPU.

### Creating the multi-hazard risk map

**Figure 6:**
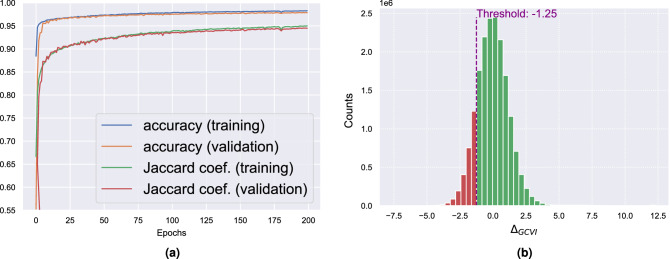
(**a**) Training and validation performances (accuracy and Jaccard coefficient) for the segmentation model $M^{tree}$. (**b**) Histogram of $\Delta _{GCVI}$. Threshold is calculated as $\mu - \sigma$ of the distribution to distinguish health from unhealthy trees.

We first train and test the segmentation model $M^{tree}$ A tree map for a portion of the city (83 $\text {km}^2$) has been manually created with a balance between vegetated and non-vegetated areas. We created a dataset by randomly extracting 10000 $80 \times 80$ pixel tiles from this area. Thus, the dataset consists of a tensor of (10000, 8, 80, 80). We recall that 8 is the number of channels in the satellite image. We then split the dataset into a training and validation set with a 80-20 % proportion, respectively, as a common practice in machine learning^37^. Accuracy and the Jaccard coefficient are used to assess the model’s performance and prevent overfitting when training comparing the segmentation output to the ground truth. Accuracy is a standard metric in classification, and it is defined as the proportion of correctly predicted pixels among the total number of pixels. The Jaccard index is one of the most used metrics in image segmentation and is defined as the ratio of intersection over the union between the prediction and the ground truth. Figure 6a shows the metrics during the training and validation procedures.

We note that the accuracy reaches a value above $95\%$ already before 20 epochs and stabilizes at $98\%$ while the Jaccard coefficient stabilizes at $95\%$. Results show that the segmentation model can detect the trees over the study area with good accuracy compared to the ground truth.

Finally, the trained model $M^{tree}$ is used to segment trees over the whole study area by splitting it into tiles of $80\times 80$ pixels, performing the prediction over each tile, and stitching the tiles back together. This way, a segmentation map for the entire area is produced. Having a working segmentation model is fundamental when extensive tree inventory databases are not available, which is the case in a lot of parts of the world that have limited information, especially regarding tree inventories.

The GCVI (Eq. 3) has been calculated from the current and historical satellite images taken in 2022 and 2021, respectively. Health is then estimated using $\Delta _{GCVI}$ and $f_{health}$ as described in the “Methodology” Section. Due to the lack of surveys or references in the study area, health cannot be directly evaluated. Nevertheless, our method relies on current studies investigating the correlation between vegetation indexes and vegetation health. Figure 6b shows the distribution of $\Delta _{GCVI}$ for the trees in the study area. The histogram confirms the Gaussian-like distribution of the empirical data. A threshold $Th = \mu -\sigma$ is defined to group the trees into healthy and unhealthy, based on the assumption that unhealthy trees have a statistically high, negative $\Delta _{GCVI}$.

**Figure 7:**
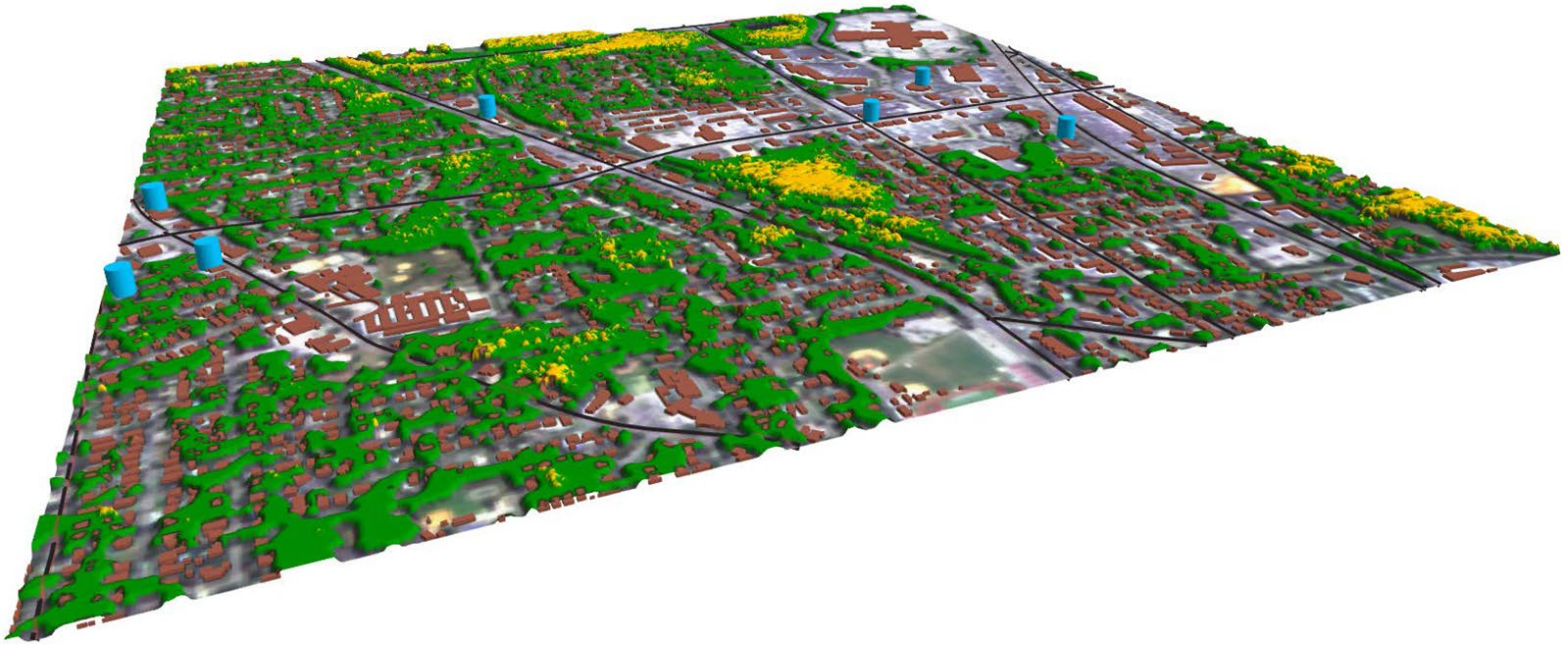
$O_{tree}^{health}$ calculated as $f_{health}(\Delta _{GCVI}) \otimes O^{tree}$. It is a three-value mask where each pixel is either 0: no-tree (not visualized), or 1: healthy tree (green), 2: unhealthy tree (yellow). The image shows additionally the buildings (brown), critical buildings locations (blue cylinders) and considered roads (black lines). The satellite images is acquired from PlanetScope^16^. The image has been created with QGIS 3.20^17^.

Figure 7 shows a portion of the output of the module $O_{tree}^{health}$ calculated as $f_{health}(\Delta _{GCVI}) \otimes O^{tree}$. The image is superimposed with roads, buildings and critical buildings.

**Figure 8:**
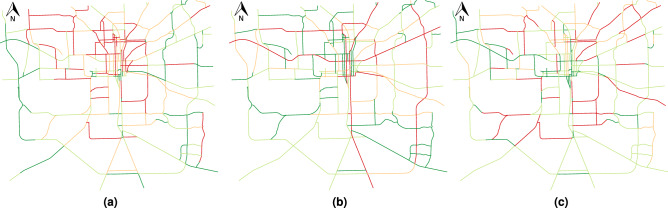
QGIS map of the study area showing roads colorized by (**a**) the number of buildings $N_b$, (**b**) betweenness centrality $\Omega$, (**c**) risk map for the study area using Eq. (10). Roads have been colorized from low (green) to high (red) values according to equal count quantile.

Figure 8a and b show the calculated values for $N_b$ and $\Omega$, respectively. Figure 8a shows the calculated values for the number of buildings per road $N_b$, showing Tallahassee’s most densely inhabited area (red segments). Figure 8b shows the calculated values for the betweenness centrality $\Omega$. Using Eq. (10) we generate a multi-hazard risk map of the area combining the aforementioned different terms. Figure 8c shows such a risk map for the whole Tallahassee. Such a map shows the roads with greater risk, meaning that they are both more prone to disruptions and a disruption significantly affects the mobility.

### Sensor placement recommendation

**Figure 9:**
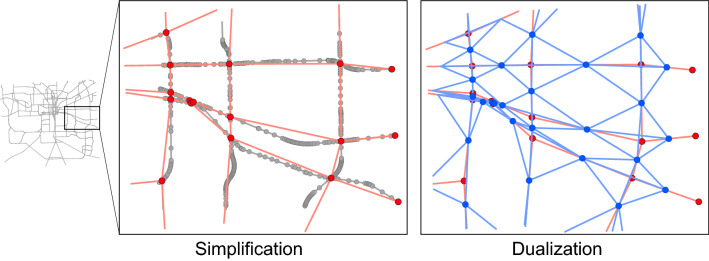
Simplification and dualization procedure of a transportation network. In the original network, nodes (e.g., intersections) that have two edges are removed because they are part of the same road. Only nodes connecting three or more edges (e.g., roads) are kept. Furthermore, nodes connected to one edge are on the boundaries of the study area and should not be removed. This leads to the red-colored graph. During the dualization process, nodes become edges and edges become nodes. This way the road attributes are stored in nodes. This leads to the blue-colored graph.

The risk map is converted into a graph, simplified and dualized as a pre-processing step. Figure 9 provides a visualization of the procedure.

**Figure 10:**
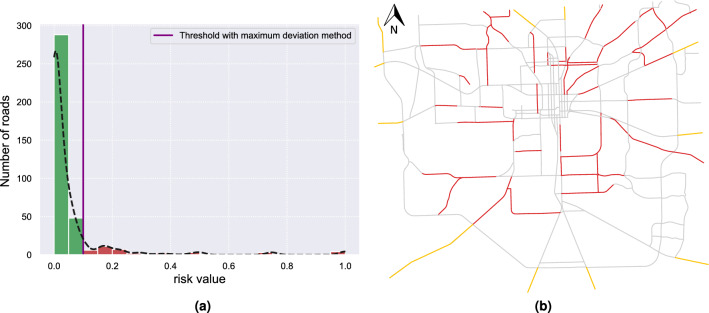
(**a**) Histogram of the risk values. The continuous distribution, shown as black solid line, has been estimated using kernel density estimation (KDE). Roads with a risk value less than the threshold are assigned to the *not at-risk* group (green), *at-risk* group (red) otherwise. (**b**) Initial monitoring subset $S_0$, defined as the union of at-risk roads (red segments) and evacuation routes (yellow segments).

As described in the “Methodology” Section, we first group the roads into *at-risk* and *not at-risk* roads based on the calculated risk function and the maximum deviation threshold. Figure 10a shows the distribution, as histogram, of the risk values and calculated threshold. Such at-risk roads are merged with the evacuation routes around the city to initialize the starting subset $S_0$ (Figure 10b). This is the set of roads that need to be monitored, setting the lower bound for the amount of sensors that need to be deployed.

**Figure 11:**
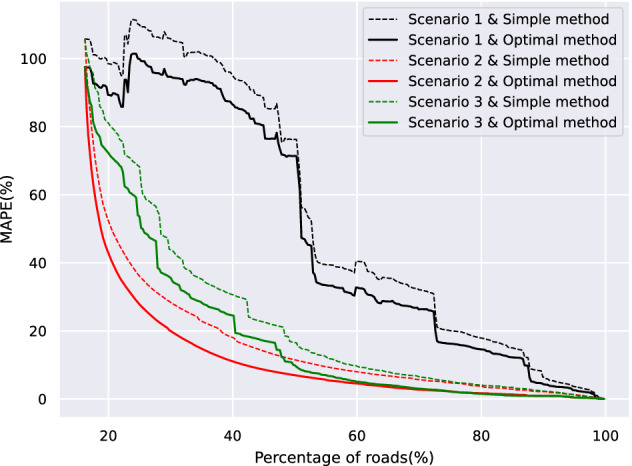
Mean absolute percentage error (MAPE) as a function of the roads covered by a sensor for scenario 1: based on risk, scenario 2: based on minimum estimation error, and scenario 3: based on combined risk and minimum estimation error.

We then simulate three different scenarios using our proposed search method (Algorithm 1) and the optimal weights found in Eq. (15). We also simulate the same scenarios with the simple average method (Eq. 11). Figure 11 shows the mean absolute percentage error (MAPE) as a function of the roads monitored over each considered scenario (scenario 1, 2 and 3) using our proposed traffic flow model (Eq. 12) and a simple node neighborhood average (Eq. 11).

In the first scenario, the selection of nodes to be added iteratively to the subset is based on risk. This means that in the search algorithm, a node with higher risk is prioritized, and the sensor is placed on it. As we see from the figure, this scenario is not optimal for situations where a low traffic estimation error is important. For instance, if a traffic estimation error of 20% error is acceptable, then we need to place sensors on 73% of the roads.

In the second scenario, the selection of nodes to be added to the subset is based on the minimum estimation error. This prioritization policy leads clearly to a lower estimation error. Similarly to the previous case, if we require at least 20% error in the traffic estimation error, then only 30% of the roads require to have a traffic sensor.

**Figure 12:**
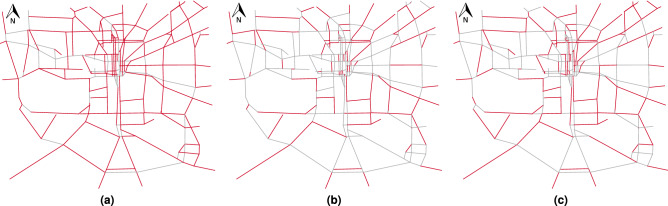
Final QGIS map output of roads that need to be monitored (red segments) by placing a traffic sensor based on: (**a**) scenario 1 with optimal weights, (**b**) scenario 2 with optimal weights, and (**c**) scenario 3 with optimal weights.

In the third scenario, a combination of the two previous scenarios is taken into consideration. Since we balance at-risk roads and traffic estimation error, we expect the results to lay between the previous two scenarios. In this case, for a 20% traffic estimation error tolerance, 40% of the roads require a traffic sensor. Finally, we note that the curves generated using the proposed traffic flow model are constantly below the one generated using the simple average. This practically means that we can achieve a smaller prediction error in the traffic data using the same number of sensors. Figure 12 shows the output of the procedure as a QGIS map based on 20% tolerance error. Depending on the prioritization policy, different roads need to be monitored by placing a traffic sensor.

### Practical considerations and broader impact

Satellite data have several advantages compared to traditional infrastructure monitoring approaches, such as ground-based and aerial inspection with helicopters or drones. Traditional ground-based techniques are known to be time-consuming, costly, and unsafe while handling the complexity associated with emergency transportation operations. Above all, there has been a tremendous drop in satellite data costs and high availability in recent years. For instance, Sentinel-1, 2, and 3 are expected to produce ca. 20 TB of free data per day^38^. This practically translates into the possibility of having many frequent, cheap, and up-to-date images, especially for large areas like an entire city. Such ”big data” are ideal fodder for data-driven (e.g., artificial intelligence or AI) approaches such as deep learning. However, there are some limitations to its application. The first limitation relates to the appropriateness of the data for the task at hand. Medium/high resolution (e.g., 3 m/px resolution used in this study) can seldom distinguish a single tree’s canopy structure and size, especially if they are packed together. As such, it is challenging to estimate single tree parameters for a detailed risk assessment. Furthermore, the estimation of tree health is also an additional source of uncertainty. In our satellite-based framework, we propose an alternative simplified model for multi-hazard risk calculation. Although the accuracy and Jaccard coefficient indicate a strong performance of our model, one of the challenges of AI-based models is the uncertainty of how they make decisions. Systematically exploring the spectrum of challenges presented when using AI-based methods for this type of application and trying to find solutions is an ongoing effort in an active community of experts at the intersection of geosciences and computer sciences/mathematics. Despite the limitations and uncertainties outlined above, we believe that our approach can serve as a complementary source for emergency management teams, not to fully replace but to be combined with the traditional roadway inspection approaches. In this paper, we considered mainly the vegetation and flood exposures as causes for roadway closures. The authors are, however, aware that this is a narrow scenario for risk assessment and during natural disasters such as those caused by hurricanes, many variables are at play. Capturing these variables in a risk assessment scenario is very challenging. For instance, urban roads are also likely to be affected by other components such as electric poles, streetlights, buildings debris etc. In general, other elements such as wildfires, landslides or avalanches should be taken into account, depending on the considered geographical area. However, vegetation and floods remain the most significant causes of road disruptions, especially in the considered study area in Tallahassee. The proposed approach can possibly be extended to other infrastructure networks, such as electricity lines and railroads, with minor modifications.

In terms of sensor placement, we propose a procedure to select and choose which roads to optimally monitor with sensors, depending on different prioritization policies (i.e., risk or traffic estimation error). Which particular strategy to be used heavily depends on the planning for the transportation system based on costs, available resources, and accepted level of traffic estimation error. These inputs may vary from one city to another, and therefore it is not possible to provide a fixed rule. However, our framework works as a general, flexible, and rapid recommendation tool for supporting municipality roadway authorities in planning and management, providing better situational awareness of the transportation network.

## Conclusions

In this paper, we propose a framework to place traffic sensors along roadways for efficient monitoring and improved situational awareness of the transportation network. The goal is to optimize the number and location of sensors to provide twofold benefits. The first targets to achieve better traffic flow surveillance and estimation along roadways during ordinary situations and second, to provide additional observability in case of natural disasters such as those caused by hurricanes. To do that, we use satellite imagery to detect the at-risk areas in the city automatically. Risk is calculated based on tree and flood exposures as well as the impact of a road closure on infrastructure accessibility. The traffic flow is modeled and estimated using a tailored graph-based estimation approach. While some uncertainties are still present, especially in the automatic risk assessment, our findings has the potential to assist planners and policymakers in better management of traffic operations. The solutions can help the city and transportation planning authorities to select the best locations to place traffic sensors for the city infrastructure, with reduced costs and resources. Furthermore, it leads to an improved situational awareness regarding locations at risk of multi-hazard. Future directions will be in testing the traffic conditions after using the proposed optimal sensory placement.

## Data Availability

The data supporting this study’s findings were provided to the authors by a commercial satellite image provider and the city of Tallahassee municipality. Still, restrictions apply to the availability of these data, which were used under license for the current study and are not publicly available. Authors will consider reasonable requests for data access from readers and discuss it with data owners for possible permissions. The corresponding author (mgaz@hvl.no) should be contacted to request the data from this study.
